# Senescent cardiac cells promote thrombus formation and impair fibrinolysis

**DOI:** 10.1007/s11357-026-02421-6

**Published:** 2026-08-01

**Authors:** Tal Shahar, Noa Ezra, Saray Chen, Olga Kryukov, Smadar Cohen

**Affiliations:** 1 Department of Biotechnology Engineering, The Avram and Stella Goldstein-Goren, Ben-Gurion University of the Negev, Beer-Sheva, 84105 Israel; 2 Regenerative Medicine and Stem Cell (RMSC) Research Center, Ben-Gurion University of the Negev, Beer-Sheva, Israel

**Keywords:** Thrombin, Senescence, Fibrinogen, Aging, Thrombosis

## Abstract

As a major contributor to cardiac diseases, thrombosis, a pathological process in which the formation of a blood clot restricts blood flow, is a leading cause of mortality worldwide. A central event in thrombosis involves thrombin-mediated cleavage of soluble fibrinogen into fibrin monomers, which polymerize into an insoluble fibrin mesh that forms the thrombus. While the risk of thrombosis increases markedly with age, the underlying cellular mechanisms linking aging and thrombus formation remain incompletely understood. Here, we identify a potential mechanism whereby cellular senescence in cardiac cells exacerbates thrombus formation via increased thrombin generation and impairs fibrinolysis, together promoting a pro-thrombotic state. We examined the pro-thrombotic phenotype of doxorubicin-induced senescence of human cardiac fibroblasts and cardiac endothelial cells. In both cell types, senescence accelerated and amplified fibrinogen polymerization. Treatment with a thrombin inhibitor reduced this polymerization and the cleavage of a thrombin-specific substrate by senescent cells, implicating thrombin as a key mediator of their pro-thrombotic phenotype. Complementary to their procoagulant effects, senescent cells also impaired thrombus resolution by suppressing plasminogen activation and downstream fibrinolysis. Gene expression analysis revealed senescence-associated upregulation of procoagulant and antifibrinolytic mediators, consistent with the observed functional effects. Finally, we show that the senescence-mediated polymerization of fibrinogen is dose dependent and attenuated by the senostatic agent, rapamycin. Collectively, these findings establish a direct mechanistic link between cellular senescence and thrombosis in cardiac cells, highlighting senescence as a potential therapeutic target in age-related thrombotic disease.

**Graphical Abstract:**

**Figure:**
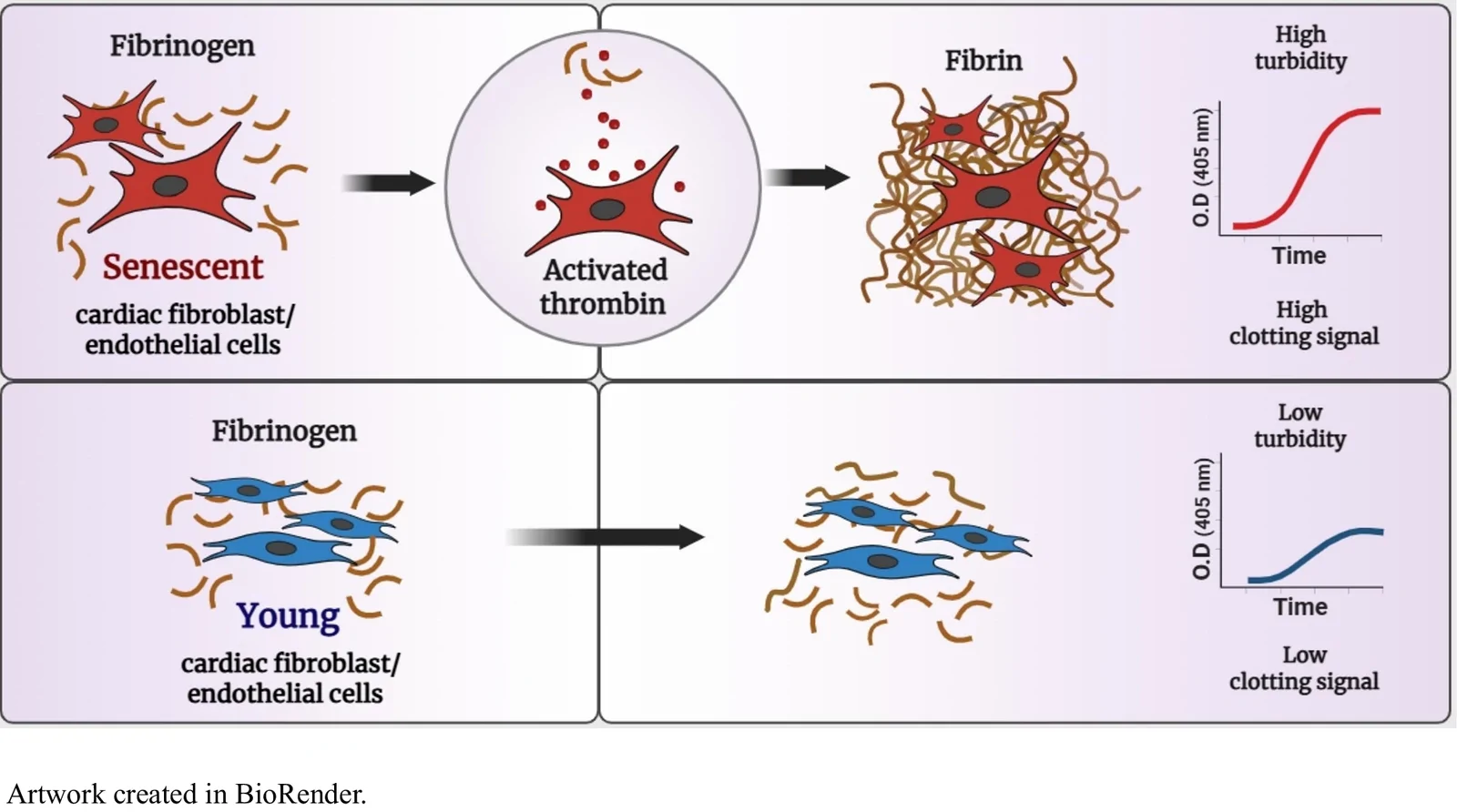

## Introduction

Cardiovascular diseases (CVDs) are the leading cause of mortality worldwide [1], with thrombosis being a key pathological mechanism underlying many of these conditions [2]. A central event in thrombosis is the enzymatic conversion of soluble fibrinogen into insoluble fibrin by thrombin, leading to intravascular clot formation and vascular occlusion. This thrombotic process underlies life-threatening complications such as myocardial infarction and ischemic stroke [3]. Given the strong age dependence of CVDs [1], there is increasing interest in how cellular aging processes such as senescence contribute to thrombosis risk.

Cellular senescence, a key hallmark of aging, is characterized by a permanent arrest of cell proliferation, while maintaining active metabolism [4]. A major feature of senescent cells is the senescence-associated secretory phenotype (SASP), a pro-inflammatory cocktail of cytokines, proteases, and growth factors. Notably, some of these secreted components influence coagulation and fibrinolysis [5]. As senescent cells accumulate with age, they contribute to tissue dysfunction across organ systems, including the cardiovascular system. Multiple lines of evidence, including animal models and human tissue studies, highlight the role of senescence in driving age-related cardiovascular pathologies [6].

Advancing age is associated with increased thrombotic risk and impaired clot resolution in CVDs [7]. While the liver is the primary source of most circulating coagulation factors [8, 9], accumulating evidence indicates that coagulation and fibrinolytic processes are also shaped locally within tissues. Human endothelial cells (ECs) and fibroblasts have been shown to express and produce coagulation components sufficient to support thrombin generation at the cell surface, highlighting a cell-associated capacity to influence fibrin formation beyond systemic factor supply [10–12].

Alongside these tissue-level mechanisms, studies of senescent cells have reported disruptions of the tight regulation of coagulation processes, such as increased secretion of hemostasis-related factors [13]. Specifically, senescent fibroblasts have been shown to secrete factors such as thrombospondin-1 (THBS1) and integrin beta-1 (ITGB1) that promote platelet activation and blood clotting. Furthermore, senescence induction with doxorubicin was found to increase blood clotting, an effect that was attenuated when senescent cells were selectively cleared. Vascular senescence has also been implicated in age-associated alterations in clot structure and stability [14]. For example, senescent ECs show reduced production and secretion of key regulators of coagulation and fibrinolysis [7]. As part of the SASP, senescent cells secrete elevated levels of plasminogen activator inhibitor-1 (PAI-1), which impairs fibrinolysis by blocking the conversion of plasminogen to plasmin, potentially stabilizing fibrin clots and promoting thrombosis [15–17]. Together, these procoagulant and anti-fibrinolytic effects enhance fibrin formation and impair its degradation, resulting in more stable and persistent clot structures.

Despite growing interest in the role of senescent cells in aging, the mechanisms linking senescence to thrombosis are not well defined. This knowledge gap is especially pronounced in cardiac cell types, where there have been few functional studies. Here, we examined how senescent cardiac fibroblasts (CFs) and ECs affect fibrinogen polymerization and degradation, focusing on the role of endogenous thrombin in these processes. Both cell types promoted accelerated and amplified fibrinogen polymerization, mediated at least in part by endogenous thrombin generation. This effect was attenuated upon treatment with the senostatic agent rapamycin. These findings reveal a functional link between cellular senescence in the cardiac environment and coagulation dynamics, with potential implications for age-related thrombotic disorders.

## Methods

### Cell cultures and induction of cellular senescence

Normal human ventricular cardiac fibroblasts (NHCFs; Lonza, CC-2904) were cultured in Fibroblast Growth Medium 3 (FGM-3) supplemented with 9% (v/v) SupplementMix (PromoCell, C-23010) and 1% (v/v) antibiotic–antimycotic solution (Capricorn, AAS-B), according to the manufacturerʼs instructions. Human aortic endothelial cells (HAECs; ScienCell, #6001) were maintained in endothelial cell medium (ScienCell, #1001) supplemented with 5% (v/v) FBS (ScienCell, #0025), 1% (v/v) endothelial cell growth supplement (ECGS; ScienCell, #1052), and 1% (v/v) penicillin–streptomycin (ScienCell, #0503). HAECs were cultured on fibronectin-coated flasks (2 µg/cm^2^; ScienCell, #8248). Both cell types were incubated at 37 °C with 5% CO₂ and were passaged at 70–80% confluency. All experiments were performed using cells in passages 3–5. To induce cellular senescence, NHCFs were treated with 250 nM doxorubicin HCl (TEVA) for 24 h, while HAECs were exposed to 200 nM doxorubicin for 48 h. These conditions were selected based on established protocols [18, 19] and internal dose–response optimization. Following treatment, cells were maintained continuously in their respective growth media for a total of 14 days to achieve cellular senescence, with medium replacement every 2–3 days. The cells were not passaged or replated during this recovery period. Untreated cells served as negative controls. For rapamycin treatment, cells were co-treated with 250 nM rapamycin (Thermo Fisher Scientific, J62473.MF) during doxorubicin exposure. This concentration was selected based on established protocols [20–22] and internal dose–response optimization. Subsequently, the culture medium was supplemented with 250 nM rapamycin at every medium change throughout the 14-day recovery period.

### SA-β-gal analysis

Cells were seeded at a 5 × 10^3^ cells/cm^2^ density and incubated overnight to allow adherence. Next, they were washed twice with warm PBS, fixed with 2% (v/v) formaldehyde (Sigma F1635) and 0.2% (v/v) glutaraldehyde (Sigma, G6257) in PBS at room temperature (RT) for 5 min, and washed with PBS twice. Then the cells were stained with a staining solution containing 1 mg/mL X-gal, 40 mM citric acid/sodium phosphate buffer (pH 6.0), 5 mM potassium ferrocyanide, 5 mM potassium ferricyanide, 150 mM NaCl, and 2 mM MgCl_2_ (Inalco Pharmaceuticals 1578–0300, Sigma 77–92-9, Sigma 567,550, Sigma P9387, Sigma P8131, Sigma 7647–14-5, Sigma S0250, respectively). The plate was sealed with Parafilm to avoid evaporation, and the cells were incubated for 12–16 h at 37°C. Subsequently, they were washed twice with PBS and incubated with NucBlue® (Thermo Fisher Scientific, R37605) for 20 min to stain nuclei and visualized using a fluorescent microscope (Olympus IX70 BF). At least three fields (accounting for a total of at least 300–1,000 cells) for every biological repeat were photographed, and the percentage of SA-β-gal positive cells was calculated relative to the number of nuclei.

### Morphology staining and immunofluorescence detection of prothrombin

Cells were seeded at 5 × 10^3^ cells/cm^2^ density and incubated at 37 °C with 5% CO₂ overnight to allow adherence. They were washed three times with PBS, fixed using 4% formaldehyde solution for 20 min, and washed three times. For morphology staining, the cells were stained immediately after fixation with phalloidin-Alexa Fluor 546 (Thermo Fisher Scientific, A22283) at RT for 1 h. They were then washed twice with PBS, incubated with NucBlue® for 20 min to stain nuclei, and visualized using an EVOS™ FL Digital Inverted Fluorescence Microscope (Life Technologies). At least three fields (accounting for a total of at least 150–700 cells) for every biological repeat were photographed. The average areas of cells and nuclei were calculated relative to the number of nuclei with ImageJ software.

For immunofluorescence, after fixation, the cells were incubated for 1 h at RT with blocking/permeabilization solution (1% BSA in PBS containing 0.1% Triton X-100) following fixation. Next, the cells were washed twice and incubated overnight at 4 °C with either a primary antibody, anti-prothrombin antibody (Abcam, ab208589), or an isotype control (Abcam, ab172730), each at a final concentration of 5.72 µg/mL in blocking/permeabilization solution. The cells were then washed twice and incubated with a corresponding secondary antibody (Abcam, ab150075) at a 1:500 dilution in blocking/permeabilization solution for 2 h at RT in the dark. Next, the cells were washed three times and incubated in PBS with phalloidin-Alexa Fluor 546 at RT for 1 h. Finally, they were washed twice with PBS and incubated with NucBlue® for 20 min to allow nuclear staining, and imaged with a confocal microscope (Zeiss LSM880 Airyscan).

### Quantitative PCR analysis

Approximately 5 × 10^5^ cells per sample were pelleted, and total RNA was extracted using a PureLink™ RNA Mini Kit (Thermo Fisher Scientific, 12183018 A), following the manufacturerʼs instructions. cDNA was synthesized from 0.5 μg of RNA using a High-Capacity cDNA Reverse Transcription Kit (Applied Biosystems, 4,368,814). The resulting cDNA was diluted tenfold and analyzed using RT-qPCR with a StepOnePlus RT-qPCR system and SYBR Green reagents (SYBR™ Green PCR Master Mix, Applied Biosystems, A46109). All reactions were performed in technical duplicates for each of the three independent biological replicates. Gene-specific primers (IDT) were used for each target, as detailed in Table 1. PUM1, recognized as a suitable housekeeping gene for senescence studies [23], was used to calculate the ΔCt. The stability of PUM1 expression was confirmed by comparing Ct values across experimental groups, which showed no significant variation. The total fold change of mRNA expression (ΔΔCt) was calculated relative to a relevant control group. For the prothrombin gene, TaqMan Gene Expression Assays (Thermo Fisher Scientific, Hs01011988_m1) and PUM1 (Thermo Fisher Scientific, Hs00472881_m1) were employed, providing greater sensitivity and successfully detecting expression changes unobserved with standard primers.

**Table 1:** Primer sequences used for RT-qPCR

Gene Symbol	Full Gene Name	Forward Primer (5ʼ−3ʼ)	Reverse Primer (5ʼ−3ʼ)
PUM1	Pumilio RNA Binding Family Member 1	CGGTCGTCCTGAGGATAAAA	CGTACGTGAGGCGTGAGTAA
CDKN1A	Cyclin Dependent Kinase Inhibitor 1 A (p21)	TCACTGTCTTGTACCCTTGTGC	GGCGTTTGGAGTGGTAGAAA
CDKN2A	Cyclin Dependent Kinase Inhibitor 2 A (p16)	ACCAGAGGCAGTAACCATGC	CCTGTAGGACCTTCGGTGAC
IL6	Interleukin 6	AGACAGCCACTCACCTCTTCAG	TTCTGCCAGTGCCTCTTTGCTG
IL8	Interleukin 8	GAGCACTCCATAAGGCACAAA	ATGGTTCCTTCCGGTGGT
IL1a	Interleukin 1 Alpha	GGTTGAGTTTAAGCCAATCCA	TGCTGACCTAGGCTTGATGA
CCL2	C–C Motif Chemokine Ligand 2	AGTCTCTGCCGCCCTTCT	GTGACTGGGGCATTGATTG
CXCL1	C-X-C Motif Chemokine Ligand 1	CATCGAAAAGATGCTGAACAGT	ATAAGGGCAGGGCCTCCT
CXCL10	C-X-C Motif Chemokine Ligand 10	GAAAGCAGTTAGCAAGGAAAGGT	GACATATACTCCATGTAGGGAAGTGA
MMP9	Matrix Metallopeptidase 9	GAACCAATCTCACCGACAGG	GCCACCCGAGTGTAACCATA
LMNB1	Lamin B1	GTGCTGCGAGCAGGAGAC	CCATTAAGATCAGATTCCTTCTTAGC
F3	Coagulation Factor III (Tissue Factor)	TTTCAGTGTTCAAGCAGTGATTCC	CTACCGGGCTGTCTGTACTCTTC
F5	Coagulation Factor V	ATGCGTGGAGAATCTGTGAC	TCTGCAAGGTTATTGACAGTG
F10	Coagulation Factor X	GCCCACTGTCTCTACCAAGC	CTTGATGACCACCTCCACCT
F11	Coagulation Factor XI	GTGGTACATTTCATTTTATTTACTTCAG	TGTCCTATTCACTCTTGGCAG
F12	Coagulation Factor XII	TGGACCAACGGACGGATG	CCCAAGGTGGAATCGAAAGTG
PLAT	Plasminogen Activator, Tissue Type (tPA)	AACAGTCACCGACAACATGC	CCATCGTTCAGACACACCAG
PLAU	Plasminogen Activator, Urokinase (uPA)	TAACGATCCCCAGTTTGGCAC	GGTCAGCAGCACACAGCATTT
PAI1	Plasminogen Activator Inhibitor 1	AAGGCACCTCTGAGAACTTCA	CCCAGGACTAGGCAGGTG
A2M	Alpha-2-Macroglobulin	GAAGGAACAGTGGTGGAATTGAC	CCGTGGTAGCATTGGAGTAATAG
CPB2	Carboxypeptidase B2	GCTGCCGGAGCGTTACAT	CATTCCTAATGACATGCCAAGCT
SERPINF2	Serpin Family F Member 2 (α2-Antiplasmin)	CAAGTTTGACCCGAGCCTTA	TACCTGGGACACGTTCCATT

### Fibrinogen polymerization and fibrinolysis assay

Cells were seeded in 96-well plates at a density of 4.4 × 10^4^ cells/cm^2^ and incubated for 24 h to allow adherence. The medium was then replaced with serum-free medium for an additional 24 h to eliminate exogenous proteins. The following day, fibrinogen (Sigma, F8630) was dissolved in 0.9% saline at 37 °C to a concentration of 40 mg/mL and diluted in equal ratios with DMEM without phenol red (Sartorius, 01–053-1A), containing 10 mM CaCl_2_ to yield final concentrations of 20 mg/mL fibrinogen and 5 mM CaCl₂. Medium was aspirated from the cells, and 100 µL of the resulting fibrinogen solution was added to each well. For positive controls, the same fibrinogen–CaCl₂ mixture was combined with thrombin (Sigma, T4648) to a final thrombin concentration of 0.5 U/mL and added to cell-free wells. To evaluate the contribution of endogenous cellular thrombin activity to fibrinogen polymerization, the thrombin inhibitor argatroban (Sigma, A0487) was added to designated wells at a final concentration of 2 µM. Additional negative controls included cell-free wells containing fibrinogen solution and cell-containing wells treated with argatroban but lacking fibrinogen, to confirm stable baseline absorbance. Fibrinogen polymerization was monitored by measuring absorbance at 405 nm every 56 s for 2 h at 37 °C using a microplate reader (Synergy H1, BioTek®).

Fibrinolysis was evaluated under similar conditions by preparing mixtures containing fibrinogen (final concentration of 20 mg/mL), thrombin (0.5 U/mL), CaCl_2_ (5 mM), and plasminogen (25 µg/mL; Abcam, ab92924). In selected wells, recombinant tissue plasminogen activator (tPA; Abcam, ab92637) was added at a final concentration of 0.1 nM to facilitate plasminogen activation. As a positive control, plasmin at a final concentration of 0.1 U/mL (Sigma, P1867) was combined with thrombin and CaCl_2_, and added to cell-free wells with fibrinogen under the same conditions. Fibrinolysis was monitored by measuring the decrease in absorbance at 405 nm every 56 s for 3 h at 37 °C using a microplate reader (Synergy H1, BioTek®).

To evaluate the effect of cell concentration on fibrinogen polymerization, cells were detached and re-suspended in 100 µL of fibrinogen–CaCl_2_ solution at three final cell concentrations: 2.5 × 10^6^, 1.5 × 10^6^, and 0.5 × 10^6^ cells/mL. The final concentrations of fibrinogen and CaCl_2_ in each mixture were 20 mg/mL and 5 mM, respectively. All conditions were prepared in a 96-well plate with 100 µL/well by combining the components. Fibrinogen polymerization was assessed by monitoring turbidity at 405 nm every 56 s for 3 h at 37 °C using a microplate reader (Synergy H1, BioTek®).

### Thrombin activity assay

Cells were seeded in 96-well plates at a density of 4.4 × 10^4^ cells/cm^2^ and incubated for 24 h. The medium was then replaced with serum-free medium for 24 h to eliminate exogenous proteins. The following day, Thrombin Substrate III (Sigma, 605,211) was prepared in DMEM without phenol red and applied to the wells at 100 µL/well and a final concentration of 0.2 mM. The thrombin inhibitor argatroban was prepared in the same medium and added to wells containing the substrate at a final concentration of 2 µM. For a positive control, thrombin was added to cell-free wells at a final concentration of 0.5 U/mL with either the fluorogenic substrate alone, or with both the substrate and argatroban. Additional negative controls, including cell-free and substrate-free wells, were included to control for background fluorescence. Fluorescence from substrate cleavage was monitored at 370/450 nm every 50 s for 2 h at 37 °C using a microplate reader (Synergy H1, BioTek®).

### Thrombin protein measurement via ELISA

Cells were seeded in T-25 flasks at a density of 9 × 10^3^ cells/cm^2^ and incubated for 24 h. Then they were washed three times with PBS containing Ca^2^⁺/Mg^2^⁺ (Sigma, D8662) to remove residual serum proteins. Next, the medium was replaced with serum-free phenol red-free DMEM, and the cells were incubated for an additional 24 h. The following day, they were washed three times with ice-cold Tris buffer (50 mM Tris–HCl, pH 7.4) to remove any remaining exogenous proteins. For lysis, 500 µL of ice-cold lysis buffer containing 50 mM Tris–HCl, pH 7.4, 150 mM NaCl, 2 mM CaCl_2,_ 1% (v/v) Triton X-100, and a protease inhibitor cocktail (Sigma, P8340) was added to each flask. The flasks were gently rocked on ice for 10 min, and the cells were detached using sterile scrapers. The lysates were transferred to microcentrifuge tubes and centrifuged at 12,000 × g for 15 min at 4°C. The resulting supernatant, representing the soluble protein fraction of the cell lysate, was collected and stored at –80°C until analysis. Thrombin protein levels were quantified using a human thrombin ELISA kit (Abcam, ab270210) and normalized to equal cell number to determine the cell-associated (lysate) thrombin reservoir.

### Cryo-SEM

A fibrinogen–CaCl_2_ solution was mixed with cells to a final cell concentration of 1.5 × 10⁶ cells/mL, and a final fibrinogen and CaCl_2_ concentration of 20 mg/mL and 5 mM, respectively. As a control, the solution was mixed with thrombin at a final concentration of 0.5 U/mL, in the absence of cells. The mixtures were then incubated overnight at 37 °C with gentle shaking to allow fibrin network formation. Subsequently, for cryo-SEM analysis, the samples were placed and sandwiched between two aluminum discs (3 mm in diameter, 100 μm in thickness each) and cryo-immobilized in a high-pressure freezing device (EM ICE, Leica). The frozen samples were then mounted on a holder under liquid nitrogen in a specialized loading station (EM VCM, Leica) and transferred under cryogenic conditions (EM VCT500, Leica) to a sample preparation freeze fracture device (EM ACE900, Leica). In that device, the samples were fractured by a rapid stroke of a cryogenically cooled knife, exposing the inner part of the sandwiched discs. After fracturing, they were etched at −90 °C for 15 min to sublime water from the sample surface and coated with 3 nm platinum. The samples were imaged in a Gemini SEM (Zeiss) by a secondary electron in-lens detector while maintaining an operating temperature of −120 °C. These measurements were done at the Ilse Katz Institute for Nanoscale Science and Technology at Ben-Gurion University of the Negev in Beer-Sheva, Israel.

### Cryo-SEM image quantification

Cryo-SEM images were analyzed using Fiji/ImageJ. At least two fields were analyzed for each biological replicate. Images were converted to 8-bit and thresholded to segment pore regions from the fibrin network. The resulting binary masks were used to quantify total pore area as a percentage of the analyzed region, and individual pore objects were analyzed to determine average pore area. To quantify fibrin mesh density, the binary pore mask was inverted to generate a fibrin-positive mask, which was then skeletonized. Mesh density was calculated as the total skeleton length normalized to the analyzed ROI area and is reported as fiber skeleton length per unit area (µm/µm^2^).

### Statistical analysis

All experiments were performed with at least three independent biological replicates unless otherwise stated. GraphPad Prism 8 software (GraphPad Software, Inc.) was used for statistical analysis. Differences between two groups were assessed using a two-tailed unpaired Studentʼs t-test. Multiple-group comparisons were performed using one-way or two-way ANOVA with Tukeyʼs post hoc test, as appropriate. Area under the curve (AUC) analysis was used to assess the overall kinetic response over time. Statistical significance was established at *p < 0.05, **p < 0.01, ***p < 0.001, ****p < 0.0001. Results are displayed as mean ± standard error of the mean (SEM).

## Results

### Doxorubicin induces senescence in CFs and ECs

**Fig. 1:**
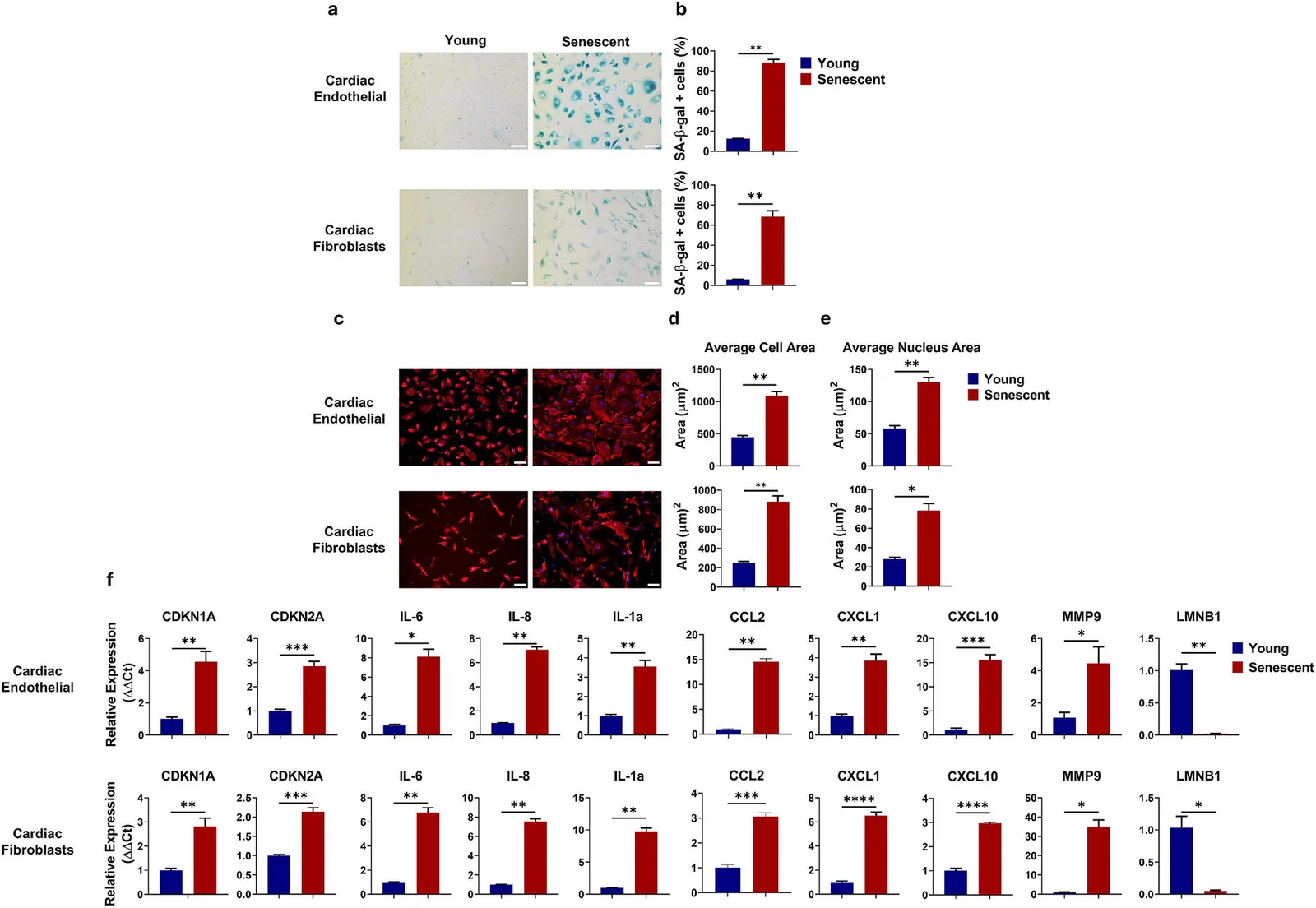
Induction of cellular senescence. Cardiac endothelial cells (ECs) were treated with 200 nM doxorubicin for 48 h, and cardiac fibroblasts (CFs) with 250 nM doxorubicin for 24 h; untreated cells served as controls (Young). **a, b** SA-β-gal activity staining and quantification (scale bar: 100 µm). **c-e** Morphological changes, including increased cell and nuclear size (scale bar: 100 µm). **f** Relative mRNA expression of senescence-associated genes (CDKN1A, CDKN2A) and selected SASP factors, normalized to PUM1. Data are presented as mean ± SEM of *n* = 3 independent biological replicates, analyzed by two-tailed Studentʼs t-test (*p < 0.05, **p < 0.01, ***p < 0.001, ****p < 0.0001)

Doxorubicin, a cardiotoxic chemotherapeutic, is a well-established inducer of premature senescence in cardiac ECs and CFs [13]. The overlap between its cardiotoxic effects and its ability to trigger senescence makes it an appropriate model for studying cardiac aging-associated diseases [24]. After doxorubicin exposure and a recovery period to allow senescence establishment, we validated the phenotype using complementary assays. SA-β-gal staining revealed a marked increase in senescent cells compared to untreated controls, with approximately 65% of ECs and 85% of CFs staining positive (Fig. 1a, b), consistent with robust senescence induction. A morphological analysis confirmed enlarged, irregularly shaped cells with expanded nuclei (Fig. 1c–e). On average, senescent ECs showed about double the cellular and nuclear area of their young counterparts, and senescent CFs displayed roughly threefold larger cell and nuclear areas.

Gene expression profiling further supported the senescent phenotype: cell-cycle inhibitors p21 (CDKN1A) and p16 (CDKN2A) were strongly upregulated, and SASP factors were broadly elevated (Fig. 1f). This included pro-inflammatory cytokines (IL-6, IL-8, IL-1α), chemokines (CCL2, CXCL1, CXCL10), and ECM remodelers (MMP9). In contrast, the nuclear lamina protein LMNB1, which is typically reduced in senescence, was downregulated [25]. Together, the integration of these multiple orthogonal markers encompassing enzymatic activity, morphological expansion, and transcriptional regulation of canonical senescence-associated genes, confirmed the successful induction of cellular senescence in both ECs and CFs in our model.

### Senescent cells accelerate fibrin formation through enhanced thrombin activity

Given the established pro-thrombotic potential of senescent cells in other contexts, and building on evidence that human fibroblasts and endothelial cells can endogenously produce coagulation proteins, we examined whether senescent ECs and CFs alter fibrin formation dynamics. A turbidimetric analysis was used to assess the effect of senescent cells on fibrinogen polymerization into fibrin, which increases turbidity and can be tracked in real time by measuring absorbance at 405 nm [26]. To ensure sufficient sensitivity for detecting relatively low levels of fibrinogen polymerization, a high concentration of fibrinogen (20 mg/mL) was utilized in these assays.

**Fig. 2:**
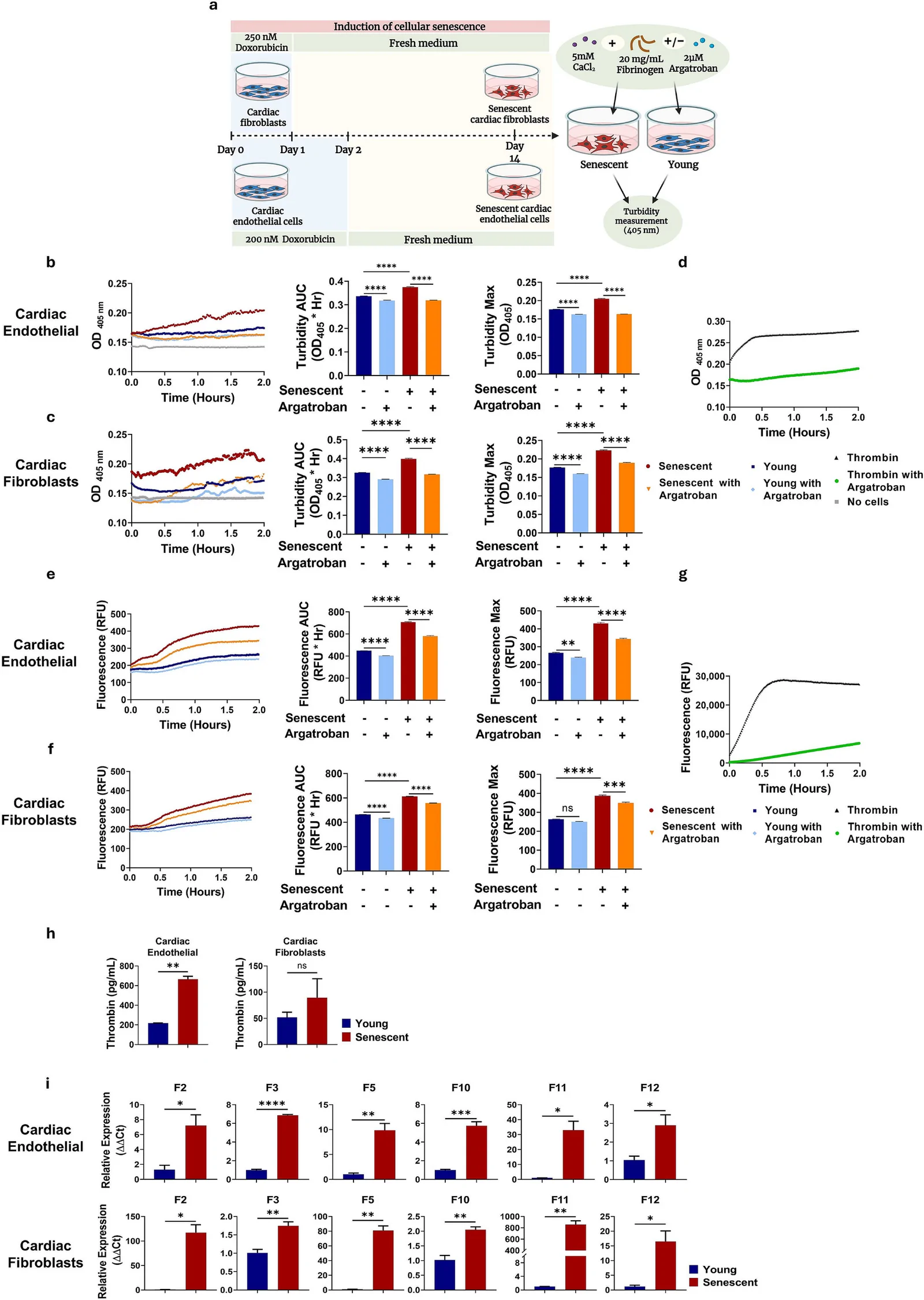
Senescent cells accelerate fibrin formation through enhanced thrombin activity.** a** Schematic representation of the experimental setup. ECs and CFs were induced to senescence using doxorubicin and seeded in 96-well plates. Fibrinogen solution was added to the cells, in the presence or absence of the thrombin inhibitor argatroban, and polymerization was assessed using turbidity measurements. **b, c** Fibrinogen polymerization kinetics (turbidity at 405 nm) in fibrinogen solution (20 mg/mL) incubated with young or senescent cells, with or without argatroban. Quantification of the AUC and maximum absorbance is shown. **d** Cell-free controls for fibrinogen polymerization using thrombin alone or thrombin + argatroban. **e, f** Thrombin activity in young and senescent cells, measured with a fluorogenic substrate (Ex/Em 370/450) ± argatroban; quantification of the AUC and maximum fluorescence are shown. **g** Cell-free controls for substrate cleavage by thrombin alone or thrombin + argatroban. **h** ELISA quantification of cell-associated thrombin protein in cell lysates normalized to cell number. **i** Relative mRNA expression of coagulation factors, normalized to PUM1. Data are presented as mean ± SEM of *n* = 3 independent biological replicates, analyzed by two-way ANOVA or two-tailed Studentʼs t-test (*p < 0.05, **p < 0.01, ***p < 0.001, ****p < 0.0001, ns = not significant). Artwork created in BioRender

Compared to young controls, the senescent ECs and CFs produced a steeper rise in absorbance and reached higher maximum values (ECs + 16%, CFs + 26%), indicating faster and more extensive fibrin network formation (Fig. 2b–d). Quantification of the AUC showed increases of 11% in the ECs and 22% in the CFs relative to the young cells. Negative controls containing only fibrinogen and CaCl₂ in the absence of cells confirmed the absence of spontaneous turbidity changes. To assess the contribution of thrombin to this effect, we repeated the assay in the presence of argatroban, a direct thrombin inhibitor that binds to the active site of thrombin [27]. Argatroban reduced polymerization in the senescent ECs by 15% (AUC) and 20% (maximum absorbance), and in the senescent CFs by 21% and 15%, respectively. The young ECs and CFs showed smaller decreases (6% in the AUC and 8% in maximum absorbance for ECs; 11% and 10%, respectively, for CFs), consistent with basal thrombin activity. These inhibitory effects indicate that thrombin activity contributes substantially to the enhanced fibrin formation observed in the senescent cells.

To directly measure thrombin enzymatic activity, we used a fluorogenic substrate selective for thrombin cleavage, which releases a fluorescent signal upon thrombin-mediated cleavage. The senescent ECs and CFs displayed a faster and more sustained fluorescence increase, reaching higher maximum values (ECs + 62%, CFs + 47%), corresponding to higher thrombin activity (AUC: + 58% in ECs, + 32% in CFs) compared to the young cells (Fig. 2e–g). Moreover, mirroring the inhibitory trend observed in the polymerization assay, argatroban decreased thrombin activity in the senescent ECs by 18% (AUC) and 20% (maximum fluorescence), and in the senescent CFs by 9% and 10%, respectively. The young ECs and CFs showed smaller reductions of 10% (AUC) and 10% (maximum fluorescence) for the ECs, and 7% and 5%, respectively, for the CFs. In line with these functional findings, an ELISA quantification of cell lysates revealed a substantial senescence-associated increase in the cell-associated thrombin levels (Fig. 2h). Senescent ECs contained substantially more thrombin protein (664 pg/mL) than the young ECs (217 pg/mL), whereas CF thrombin levels remained low (50–80 pg/mL) in both conditions despite the functional differences observed.

Furthermore, the senescent cells showed a coordinated upregulation of multiple coagulation-related genes compared to young controls (Fig. 2i). The expression of initiator clotting genes F3 and F12 was elevated, with F12 increasing 16-fold in the CFs and F3 sevenfold in the ECs. Moreover, genes promoting thrombin generation (F5, F10, F11) and the thrombin precursor gene F2 increased significantly. For example, F11 increased 800-fold in the CFs and 30-fold in the ECs, while F2 rose 120-fold in the CFs and sixfold in the ECs. While these large fold changes partly reflect the near-undetectable baseline expression of these factors in young cells, the transition to moderate expression levels in the senescent state (e.g., Ct ~ 30–33) indicates a significant biological shift. Additionally, these patterns indicate that while both senescent ECs and CFs shift toward a procoagulant transcriptional profile, CFs exhibit higher relative expression changes for several key factors, representing a more pronounced departure from their young baseline.

Overall, these findings indicate that senescent ECs and CFs enhance fibrin formation, in part through increased thrombin activity, supported by transcriptional upregulation of key coagulation factors.

### Senescent cells exhibit reduced fibrinolytic activity

**Fig. 3:**
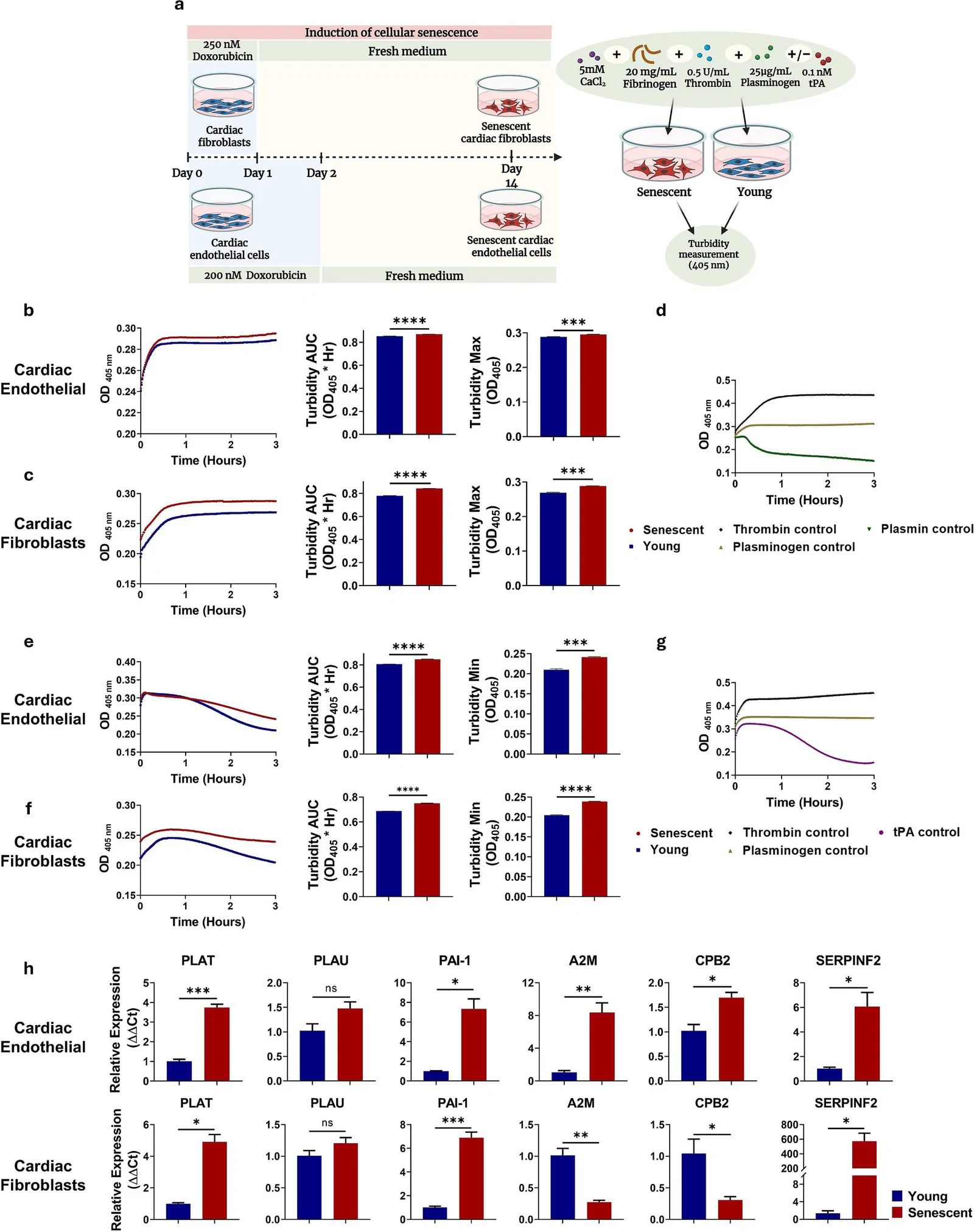
Senescent cells exhibit reduced fibrinolytic activity.** a** Schematic representation of the experimental setup. ECs and CFs were induced to senescence using doxorubicin and seeded in 96-well plates. Fibrinogen solution containing thrombin and plasminogen was added to the cells, in the presence or absence of tPA, and clot formation and fibrinolysis were assessed using turbidity measurements.** b, c** Clot formation and stability (absorbance at 405 nm) with young or senescent cells in the presence of exogenous thrombin and plasminogen (no exogenous tPA). Quantification of the AUC and maximum absorbance is shown. **d** Cell-free controls for clot formation and fibrinolysis using thrombin alone, thrombin + plasminogen, or thrombin + plasmin. **e, f** Clot formation and fibrinolysis kinetics (absorbance at 405 nm) with young or senescent cells in the presence of exogenous thrombin, plasminogen, and tPA. Quantification of the AUC and minimum absorbance is shown. **g** Cell-free controls for clot formation and fibrinolysis using thrombin alone, thrombin + plasminogen, or thrombin + plasminogen + tPA. **h** Relative mRNA expression of antifibrinolytic genes, normalized to PUM1. Data are presented as mean ± SEM of *n* = 3 independent biological replicates, analyzed by two-tailed Studentʼs t-test (*p < 0.05, **p < 0.01, ***p < 0.001, ****p < 0.0001, ns = not significant). Artwork created in BioRender

Following the observation that senescent cells accelerate fibrin formation, we next examined whether they also influence fibrin breakdown. Specifically, to measure whether the cells activate plasminogen, clots were formed by adding exogenous thrombin and plasminogen to fibrinogen solutions, in the absence of an exogenous plasminogen activator, and in the presence of either young or senescent ECs and CFs. A similar turbidimetric assay was then used to assess fibrinolysis, the enzymatic process that degrades fibrin clots, by monitoring the decline in absorbance at 405 nm. In this experiment, after clot formation, neither the young nor senescent cells induced substantial turbidity loss, suggesting minimal endogenous plasminogen activation (Fig. 3b–d).

To evaluate fibrinolysis when plasminogen activation is externally induced, and given the known upregulation of plasminogen activator inhibitors in senescent cells (e.g., PAI-1), we repeated the experiment with the addition of tissue plasminogen activator (tPA). Under these conditions, turbidity decreased progressively in all groups, consistent with fibrin degradation (Fig. 3e–g). However, the rate and extent of clot breakdown were reduced in the senescent cells. Notably, the senescent ECs and CFs exhibited a slower decline in absorbance and maintained higher turbidity over time compared with their young counterparts, with AUCs elevated by 5% in the ECs and 9% in the CFs, indicating prolonged clot persistence. The minimum absorbance reached was also higher (+ 15% in ECs, + 17% in CFs), further supporting impaired fibrinolytic capacity.

To further elucidate the molecular basis for this anti-fibrinolytic phenotype, we measured the expression of key fibrinolysis-related genes (Fig. 3h). We found that several potent fibrinolysis inhibitors were strongly upregulated in the senescent cells. For instance, SERPINF2 (α2-antiplasmin, which neutralizes plasmin) increased fivefold in the ECs and 500-fold in the CFs. Similar to the coagulation genes described above, this large fold change is amplified by near-undetectable baseline expression in young cells; nevertheless, the transition to moderate expression levels in the senescent state (Ct ~ 30) represents a significant biological shift. PAI-1, the major inhibitor of tPA and uPA, also rose significantly (eightfold in the ECs and sixfold in the CFs). Other inhibitors, such as A2M and CPB2, were only slightly elevated. In contrast, fibrinolysis activators showed only a modest change. PLAT (tPA) was moderately upregulated (fourfold in the ECs, fivefold in the CFs), and PLAU (uPA) remained unchanged. This imbalance of marked increases in key inhibitors coupled with more limited changes in activators likely underlies the reduced fibrin clearance that was observed in the functional assays.

Collectively, these functional and transcriptional data demonstrate that senescent ECs and CFs impair fibrin breakdown through significant upregulation of anti-fibrinolytic mediators, reinforcing a perpetuating pro-thrombotic microenvironment.

### Cell number and rapamycin modulate fibrin polymerization and structure

**Fig. 4:**
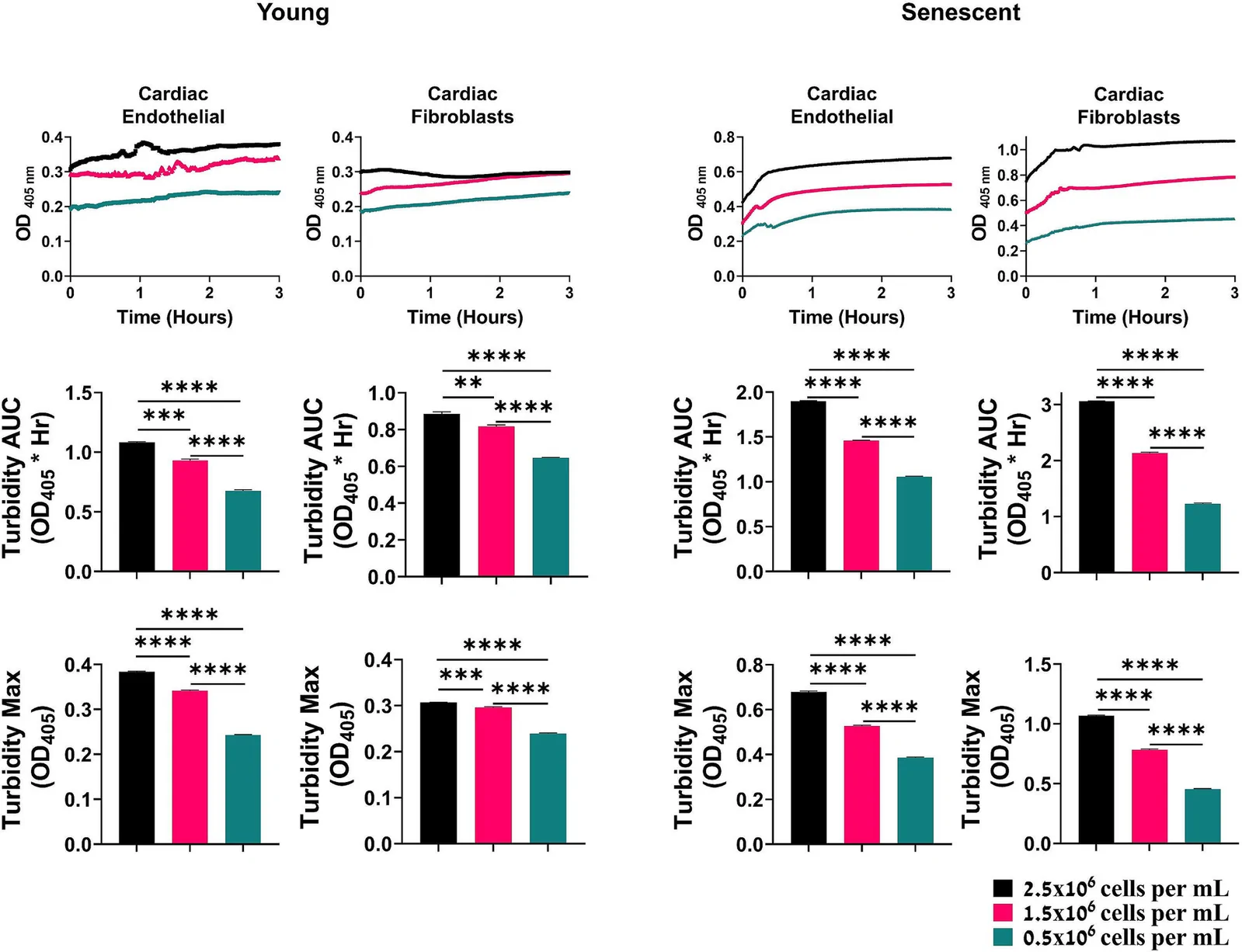
Higher cell numbers accelerate fibrin network formation. Turbidity assays (absorbance at 405 nm) of fibrinogen solution mixed with varying numbers of senescent or young cells (2.5 × 10^6^ cells/mL, 1.5 × 10^6^ cells/mL, and 0.5 × 10.^6^ cells/mL). Quantification of the area under the curve (AUC) and maximum absorbance is shown. Data are presented as mean ± SEM of *n* = 3 independent biological replicates, analyzed by one-way ANOVA (**p < 0.01, ***p < 0.001, ****p < 0.0001, ns = not significant)

To examine whether cell concentration influences fibrin formation—a variable that could model the impact of increased senescent cell burden on thrombotic risk—we measured fibrin polymerization kinetics in a 3D cell-laden fibrin clot at varying cell densities. Turbidity at 405 nm increased over time in all conditions, indicating ongoing fibrin polymerization (Fig. 4). In both the ECs and CFs, higher cell concentrations were associated with greater AUC and maximum absorbance values, with both young and senescent cells showing a consistent dose-dependent trend. At each tested concentration, the senescent cells exhibited higher absorbance parameters than their young counterparts. In the senescent ECs, the highest density (2.5 × 10⁶ cells/mL) yielded 80% higher AUC values and 75% higher maximum absorbance compared with the lowest density (0.5 × 10⁶ cells/mL). The senescent CFs exhibited an even greater increase, with AUC and maximum absorbance values higher by 148% and 134%, respectively. In the young ECs, the highest density yielded 60% higher AUCs and 58% higher maximum absorbance, while the young CFs showed increases of 37% in AUC values and 28% in maximum absorbance. These trends align with our previous findings that senescent cells display enhanced procoagulant activity per cell, potentially reflecting higher secretion of procoagulant factors or thrombin activity.

**Fig. 5:**
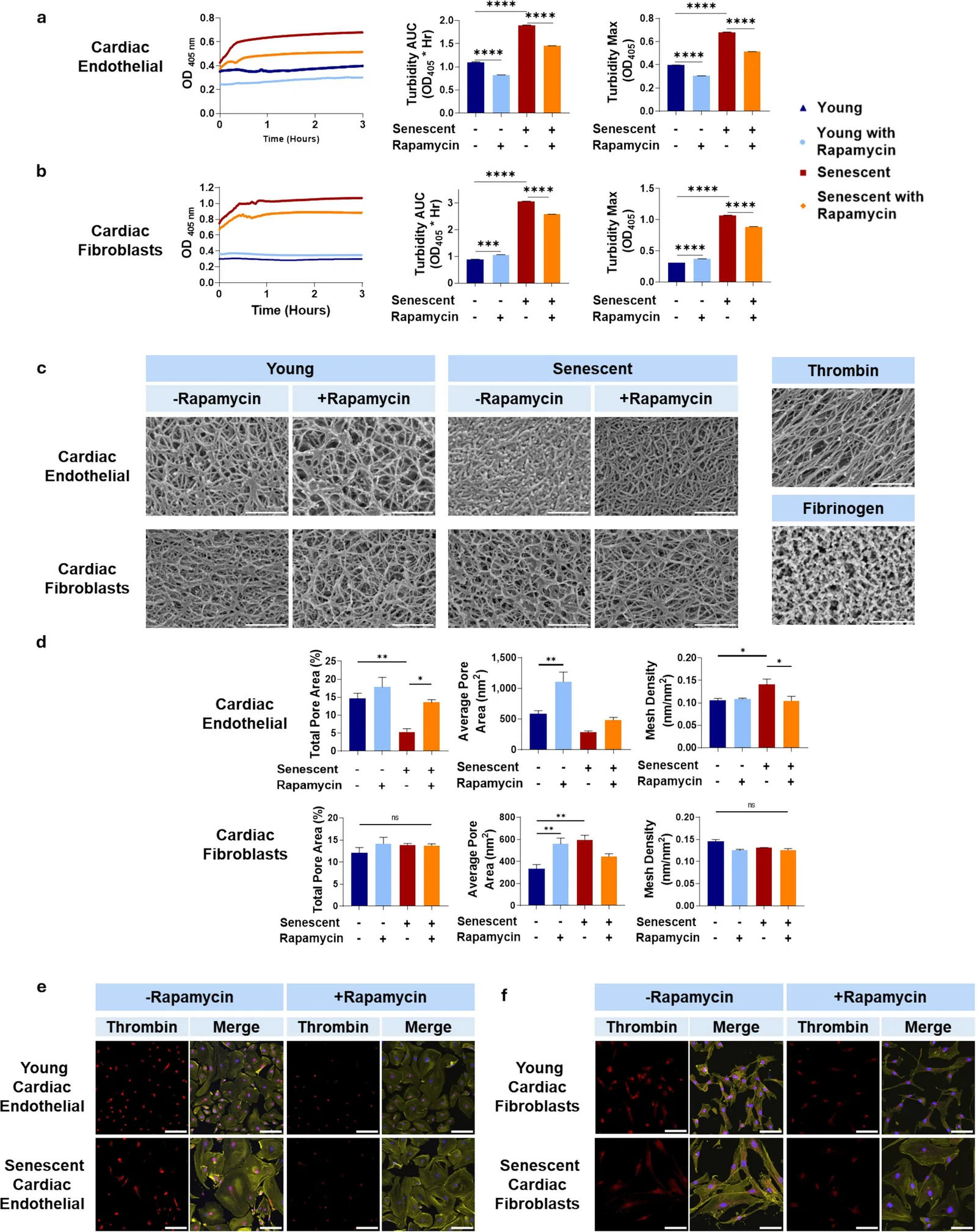
Rapamycin attenuates fibrinogen polymerization and alters fibrin structure. **a, b** Turbidity assays of fibrinogen solution mixed with senescent or young cells ± rapamycin (250 nM). Quantification of the AUC and maximum absorbance is shown. **c** Cryo-SEM images showing cell-associated fibrin structure in the presence of fibrinogen solution, comparing senescent and young cells ± rapamycin. Scale bar, 400 nm. **d** Quantification of cryo-SEM images, including total pore area, average pore area, and fibrin mesh density. Mesh density was calculated as total skeleton length normalized to ROI area. **e, f** Immunofluorescence staining for prothrombin in senescent and young cells ± rapamycin. Scale bar, 200 µm. Data are presented as mean ± SEM of *n* = 3 independent biological replicates, analyzed by two-way ANOVA (*p < 0.05, **p < 0.01, ***p < 0.001, ****p < 0.0001, ns = not significant)

To assess whether this procoagulant effect can be attenuated pharmacologically, we treated senescent cells with 250 nM rapamycin throughout the 14-day senescence induction period. Rapamycin reduced fibrin polymerization kinetics in both the ECs and CFs compared with untreated senescent controls, with a decrease in AUCs by 23% and maximum absorbance by 24% in the ECs, and by 15% and 16% in the CFs, respectively (Fig. 5a, b). However, polymerization levels remained greater than in the young cells, indicating an incomplete reversal of the senescent phenotype. Cryo-SEM imaging and quantitative image analysis revealed that senescent ECs formed denser fibrin networks than young ECs, characterized by reduced total pore area, smaller average pore area, and increased mesh density (Fig. 5c, d). Rapamycin treatment partially reversed these EC-associated structural changes, increasing pore area and reducing mesh density toward the young-cell phenotype. In contrast, CF-derived clots showed altered average pore area, but no significant change in total pore area or mesh density, suggesting a more heterogeneous structural response despite their higher polymerization kinetics. Representative immunofluorescence images of prothrombin staining are presented as qualitative visual observations alongside the functional and structural analyses (Fig. 5e, f).

These results demonstrate that fibrin polymerization is enhanced in a cell density-dependent manner in both young and senescent cardiac cell types, with a more pronounced effect in senescence. They further show that rapamycin can partially suppress this procoagulant activity, suggesting that the pro-thrombotic contribution of senescent cells may be at least partly mitigated through pharmacological interventions targeting the senescent phenotype.

## Discussion

Although aging is strongly associated with increased thrombotic risk, the underlying cellular mechanisms remain unclear. Prior evidence linking thrombosis to cellular senescence has focused primarily on paracrine SASP factors that enhance platelet production and activation or modulate immune cells [7, 13]. In this study, we showed that senescent cardiac cells significantly accelerate and amplify fibrinogen polymerization dynamics, suggesting that cellular senescence within the cardiac niche may actively shape the pro-thrombotic environment of aging.

We compared the fibrinogen polymerization capacity of senescent CFs and ECs to that of their young counterparts (Fig. 2). In both cell types, polymerization kinetics were accelerated, reaching peak absorbance significantly faster than in the young cells. Furthermore, fibrinogen polymerized by the senescent cells reached a higher maximum absorbance. Of note, in assays with only purified thrombin and fibrinogen, higher maximum absorbance typically indicates lower thrombin concentrations, because a slower reaction rate allows more time for the lateral aggregation of monomers into thicker, more light-scattering fibers. However, in plasma-based assays, maximum absorbance correlates directly with thrombin concentration, as molecular crowding favors network density over fiber thickness [28]. Because clot turbidity depends on both thrombin kinetics and the surrounding macromolecular environment, the higher maximum absorbance we observed is consistent with increased thrombin activity in our cell-containing system. This interpretation is supported by the marked argatroban sensitivity and by the thrombin-substrate assay. Notably, the slower (1–2 h) kinetics in our assay likely reflect gradual, cell-driven thrombin activity rather than the rapid, injury-triggered coagulation seen in vivo—potentially modeling the chronic, low-grade prothrombotic shift associated with aging.

Complementing these kinetic observations, additional structural and biochemical evidence supports increased thrombin activity in the senescent cells. Senescent cells of both types exhibited higher thrombin activity, as measured by increased cleavage of a thrombin-specific substrate, and reduced activity when treated with the direct thrombin inhibitor argatroban, similar to its effect in the polymerization assays (Fig. 2). Cryo-SEM analysis further showed that senescence altered fibrin structure in a cell type-specific manner (Fig. 5). EC-derived clots displayed a denser mesh architecture, with reduced pore area and increased mesh density, consistent with higher thrombin-associated polymerization activity. These structural features resemble clots formed under high-thrombin conditions, whereas low-thrombin polymerization typically produces less dense networks with larger pores [29, 30]. In contrast, senescent CF-derived clots showed increased average pore area without a significant change in total porosity or mesh density, suggesting a more heterogeneous fibrin architecture that may reflect post-polymerization remodeling or protease secretion (such as MMPs) by senescent fibroblasts.

Despite the indications of thrombin activity in the functional assays in both cell types, thrombin protein levels were elevated only in the senescent ECs, but not in the senescent CFs, as detected by ELISA (Fig. 2h). This discrepancy may reflect a requirement of the CFs for context-dependent activation that was absent in the serum-free lysates used for ELISA. This type of activation may be mediated by fibrinogen-binding receptors, such as integrin αvβ3, which is expressed by fibroblasts and capable of triggering downstream activation and differentiation into myofibroblasts upon substrate binding [31, 32]. While absolute thrombin protein levels measured in lysates remained in the pg/mL range, the known capacity of these cell types to assemble prothrombinase complexes at the cell surface suggests that catalytic activity may be highly localized [10, 11]. This cell-associated reservoir may thus allow for significant functional impact on fibrinogen polymerization despite the absence of liver-scale bulk concentrations.

Strikingly, the polymerization effect of the senescent cells was dose dependent, with higher cell concentrations inducing faster and greater polymerization (Fig. 4). This in-vitro dose dependence indicates that the thrombin-mediated fibrinogen polymerization in our system is sensitive to senescent cell burden, and may represent one cellular-level component contributing to the elevated thrombotic risk observed in vivo with increasing senescence [7]. Notably, these dose-dependence assays revealed higher maximum absorbance in the senescent CFs compared to the senescent ECs. This aligns with their markedly greater upregulation of procoagulant genes such as F2 (prothrombin) and F11 (factor XI) (Fig. 2i).

The observation that the procoagulant shift upon senescence is more pronounced in CFs than in ECs is noteworthy, particularly as ECs directly contact soluble fibrinogen and normally govern hemostasis. A plausible explanation is that young ECs constitutively express and tightly regulate many components of the coagulation cascade [11], maintaining an activated but balanced hemostatic profile. Consequently, their senescent state may represent a constrained shift on an already responsive baseline. In contrast, young fibroblasts typically maintain extracellular matrix (ECM) homeostasis with little involvement in initiating the coagulation process. Upon entering senescence, however, fibroblasts acquire a pro-inflammatory and pro-fibrotic SASP [13], and may disproportionately upregulate pro-thrombotic mediators. Such findings underscore that the senescence-associated procoagulant phenotype is likely cell-type specific, reflecting the magnitude of change from a young to a senescent state.

Beyond these cell-specific variations, the robust increase in polymerization and the coordinated upregulation of multiple factors within the coagulation cascade in these cardiac senescent cells suggest the emergence of an ectopic, cell-driven procoagulant phenotype. Characterized by the expression of factors typically enriched in hepatic synthesis, such as F11 and F2 [8, 9], this ectopic shift is additionally elucidated by the transition from a near-silent baseline in young cells, as seen in our gene expression assays. However, because several coagulation-related transcripts were near-undetectable in young cells, the magnitude of these fold changes should be interpreted cautiously and viewed primarily as evidence of a senescence-associated transcriptional shift rather than as a strictly proportional measure of pathway activation. Despite this limitation, the intrinsic nature of this localized activity is further supported by the uniform serum starvation performed prior to all functional and protein assays to deplete exogenous factors, indicating that the observed activity is likely independent of systemic factor uptake. Ultimately, this shift may indicate that the cardiac niche in aging does not merely react to systemic changes, but may actively contribute to local thrombotic events. To that end, elucidating the definitive molecular mechanisms underlying this cellular source of thrombin presents a compelling direction for future investigations, promising deeper insights into the broader landscape of vascular aging.

This study also shows that the senescent phenotype not only amplifies fibrinogen polymerization, but also delays fibrinolysis (Fig. 3). Exogenous plasminogen addition failed to induce fibrinolysis, indicating that endogenous activation of plasminogen is insufficient in our system. In contrast, the addition of exogenous tPA (which activates plasminogen) triggered clot lysis in both young and senescent cell conditions, although clots formed by the senescent cells were more resistant to breakdown. This suggests that senescent cells impair fibrinolysis by limiting plasminogen activation and/or by inhibiting plasmin itself. Consistent with these results, both senescent CFs and ECs upregulated antifibrinolytic mediators, including PAI-1, which inhibits tPA, alongside cell-type specific increases in direct plasmin inhibitors such as SERPINF2 in CFs and A2M and CPB2 in endothelial cells. Similarly to the upregulated procoagulant genes discussed above, the very large fold change observed for SERPINF2 in senescent CFs should be interpreted in the context of its near-undetectable baseline expression in young cells. Nevertheless, these expression profiles suggest that senescent CFs and ECs may promote anti-fibrinolytic responses through complementary and potentially synergistic mechanisms.

Rapamycin, an mTOR inhibitor, is a well-established senomorphic that can suppress the SASP and prevent paracrine senescence both in vitro and in vivo [33]. We hypothesized that senescence-associated increases in coagulation-related activity, particularly thrombin-associated activity, may represent a SASP-linked phenotype. Under rapamycin treatment, a reduction in fibrinogen polymerization was observed in both the senescent and young cells, with more pronounced effects at higher cell concentrations and in the senescent cells (Fig. 5). Fibrils polymerized by the rapamycin-treated senescent cells closely resembled those of the young cells, as observed by cryo-SEM imaging. Our findings show that the senescence-associated increase in fibrinogen polymerization is rapamycin sensitive, supporting its possible classification as part of the SASP. However, although rapamycin reduced fibrinogen polymerization in our system, the prothrombin immunofluorescence images were not acquired as a quantitative dataset and should be interpreted only as representative qualitative observations. Therefore, whether rapamycin directly reduces cell-associated thrombin protein levels or thrombin activity will require dedicated quantitative analysis under rapamycin-treated conditions in future mechanistic studies. Nonetheless, these results highlight the potential of senomorphic approaches to reduce senescence-associated pro-thrombotic changes.

It should be acknowledged that the coagulation cascade is highly complex and involves many interacting factors not fully captured in our in-vitro system, including the presence of platelets and circulating coagulation factors typically supplied by the liver [34]. Under these simplified conditions, our functional assays serve as a readout for cell-driven fibrin assembly, rather than a full recapitulation of systemic physiological coagulation. Because this isolated model lacks the systemic components that normally amplify thrombin generation and contribute to clot structure, a higher substrate concentration (20 mg/mL) was required to achieve a measurable signal from the localized, cell-associated thrombin activity and to clearly resolve the kinetic differences between experimental groups. While such concentrations may be relevant to extreme pathological states associated with hyperfibrinogenemia and chronic inflammation, they may not fully represent the dynamics occurring at standard physiological ranges (~ 2–4 mg/mL) [35, 36]. Consequently, further studies utilizing plasma-based models and physiological fibrinogen concentrations will be necessary to validate these findings within the broader context of the systemic hemostatic environment.

Beyond these systemic considerations, other mechanisms may contribute to the pro-thrombotic phenotype of senescent cells. While our assays show a clear thrombin dependency through the use of a specific thrombin substrate and a thrombin inhibitor, other factors may also contribute to the ability of the senescent cells to polymerize fibrinogen in a thrombin-independent manner. For example, Tissue Transglutaminase (TG2), which is often upregulated in aging and senescent cells, can directly cross-link fibrinogen into a stable matrix [37, 38]. Furthermore, while aortic endothelial cells are a well-established model for human vascular dysfunction [39, 40], future research could extend these findings by exploring site-specific variations in other vascular beds, as well as the contributions of other dominant cardiac populations such as cardiomyocytes. Additionally, comparative studies with hepatocytes, the primary systemic source of coagulation factors [34], could further elucidate the relative impact of local versus systemic senescence on thrombotic risk. Nonetheless, by isolating these direct cell-mediated effects in canonical vascular and fibroblast models, our findings provide direct evidence that senescent CFs and ECs can promote pro-thrombotic clot dynamics in vitro.

Collectively, our results suggest that senescent cardiac cells actively shape clot dynamics by enhancing thrombin-dependent fibrinogen polymerization, while simultaneously impairing fibrinolysis and clot resolution. By demonstrating that these pro-thrombotic effects scale with cell density, our findings suggest that the impact of cellular senescence on thrombosis is likely progressive, intensifying as the chronic senescent burden accumulates over time. This study thus elucidates a cellular mechanism by which senescence can promote age-related thrombosis and highlights senescence as a potential therapeutic target in age-related thrombotic disease.

## Data Availability

The data that support the findings of this study are available from the corresponding author upon reasonable request.
